# Phenotypic antimicrobial susceptibility and genomic resistance determinants in canine otitis-associated *Pseudomonas aeruginosa* isolates

**DOI:** 10.3389/fvets.2026.1902036

**Published:** 2026-07-21

**Authors:** Mercédesz Adrienn Veres, Zsófia Anna Tóth, Enikő Illés, Patrik Mag, Eszter Kaszab, Enikő Fehér, Ádám Kerek, Ákos Jerzsele

**Affiliations:** 1 Department of Pharmacology and Toxicology, University of Veterinary Medicine Budapest, Budapest, Hungary; 2 National Laboratory of Infectious Animal Diseases, Antimicrobial Resistance, Veterinary Public Health and Food Chain Safety, University of Veterinary Medicine Budapest, Budapest, Hungary; 3 Department of Bioinformatics, One Health Institute, Faculty of Health Sciences, University of Debrecen, Debrecen, Hungary; 4 Department of Microbiology and Infectious Diseases, University of Veterinary Medicine Budapest, Budapest, Hungary; 5 National Laboratory of Virology, Szentágothai Research Centre, University of Pécs, Pécs, Hungary

**Keywords:** aminoglycosides, antimicrobial resistance, canine otitis externa, fluoroquinolones, MIC, one health, *Pseudomonas aeruginosa*, whole-genome sequencing

## Abstract

**Background:**

*Pseudomonas aeruginosa* is a clinically important opportunistic pathogen in canine otitis externa, particularly in chronic, recurrent or treatment-refractory cases. Its low outer-membrane permeability, biofilm-forming capacity, efflux systems and ability to acquire additional resistance determinants complicate empirical therapy and make local surveillance essential.

**Methods:**

This study integrated broth microdilution-based minimum inhibitory concentration (MIC) testing with whole-genome analysis to characterize antimicrobial susceptibility patterns and genomic resistance determinants in canine otitis-associated *P. aeruginosa* isolates from Hungary. A total of 110 isolates were tested against 14 antimicrobial or antiseptic agents. Official clinical breakpoints and European Committee on Antimicrobial Susceptibility Testing (EUCAST) epidemiological cut-off values were applied where available, while agents lacking validated interpretive criteria were evaluated descriptively. A phenotypically selected subset of 70 isolates, chosen to cover the major MIC ranges, resistance profiles and modified MAR-index spectrum observed in the full collection, was subjected to long-read sequencing, and all 70 genome assemblies were included in comprehensive antibiotic resistance database (CARD)-based resistance gene screening, multi-locus sequence typing (MLST) and genome quality assessment.

**Results:**

High frequencies of elevated MICs were detected for several clinically relevant agents. The highest proportions above interpretive thresholds were observed for enrofloxacin (109/110, 99.1%), marbofloxacin (99/110, 90.0%), piperacillin-tazobactam (75/110, 68.2%), ceftazidime (71/110, 64.5%) and tobramycin (62/110, 56.4%). The median modified multiple-antimicrobial-resistance index, calculated only from agents with interpretable resistance thresholds, was 0.56. Genomic analysis showed a broad intrinsic resistance background dominated by efflux-associated determinants and recurrent aminoglycoside, beta-lactam and fluoroquinolone-associated genes, including *APH(3′)-IIb*, Mex-family efflux components, Opr-associated transporters, *arnA*, *basS*, PDC/OXA beta-lactamases and *crpP* in a subset of isolates.

**Conclusion:**

These findings support the need for culture- and MIC-guided therapy in canine *P. aeruginosa* otitis and support the value of including companion-animal clinical isolates in national and regional antimicrobial resistance surveillance frameworks.

## 1 Introduction

Otitis externa is among the most frequent disorders encountered in small-animal practice. Although the clinical syndrome is multifactorial, involving primary, predisposing and perpetuating factors, bacterial overgrowth and dysbiosis are central determinants of chronicity, pain, discharge, epithelial proliferation and therapeutic failure. *Pseudomonas aeruginosa* is especially relevant in this context because it is frequently associated with chronic suppurative otitis, ulceration, marked inflammation and recurrent disease. In canine ears, the organism is rarely a simple primary cause; rather, it is an opportunistic pathogen that expands when the local microenvironment, antimicrobial exposure, moisture, inflammation, biofilm formation and ear canal anatomy favor its persistence (1–4).

The therapeutic relevance of *P. aeruginosa* in canine otitis is disproportionate to its frequency. The organism combines low outer-membrane permeability, inducible beta-lactamase activity, multiple resistance-nodulation-division efflux pumps, aminoglycoside-modifying enzymes, adaptive biofilm-mediated tolerance and a capacity for target-site mutations (5). These features make susceptibility unpredictable and can allow rapid loss of response during or after antimicrobial treatment. Topical therapy can achieve high local drug concentrations, but systemic treatment may be needed when deeper tissues or otitis media are involved, and topical failure remains common when biofilm, heavy exudate, stenosis, ulceration or insufficient cleaning prevent drug penetration (1, 4–12).

Antimicrobial susceptibility testing is therefore a central component of rational management in clinically relevant *Pseudomonas* otitis. This is particularly important for fluoroquinolones, aminoglycosides, antipseudomonal beta-lactams and polymyxins, which remain important either in companion-animal otology, in severe infections, or in human medicine. Several of these compounds fall into categories requiring restriction or careful use in veterinary practice, and their continued usefulness depends on targeted therapy and surveillance rather than repeated empirical exposure (12–16).

Most veterinary studies on canine otitis-associated *P. aeruginosa* have been either phenotypic or genomic. Phenotypic minimum inhibitory concentration (MIC) testing provides the clinically actionable readout, but it does not explain why isolates show elevated MICs. Conversely, genome screening can identify resistance determinants, mobile genetic elements, sequence types and potential transmission markers, but gene presence alone does not guarantee phenotypic expression. A combined phenotypic-genomic approach is therefore needed to avoid overinterpretation of either data layer and to distinguish core intrinsic resistance background from acquired or potentially mobile resistance determinants (12, 17–21). This approach is consistent with recent Hungarian veterinary literature emphasizing that phenotypic susceptibility testing and genotypic resistance-gene detection provide complementary information for interpreting antimicrobial resistance in veterinary bacterial pathogens (22). In Hungary, whole-genome sequencing (WGS)-based resistome analysis has already been applied to veterinary *E. coli* isolates, supporting the value of genome-level antimicrobial resistance (AMR) surveillance in animal-associated bacterial populations (23).

The primary objective of this study was to integrate phenotypic antimicrobial susceptibility testing with whole-genome-based resistance determinant analysis in canine otitis-associated *P. aeruginosa* isolates. Using a 110-isolate MIC-tested collection and a phenotypically selected 70-isolate WGS subset, we aimed to define clinically relevant susceptibility patterns and place them into a genomic and One Health surveillance context.

## 2 Materials and methods

### 2.1 Study design and isolate collection

This was an observational laboratory study of archived bacterial isolates from canine otitis externa cases submitted to a veterinary microbiology laboratory. The phenotypic dataset comprised 110 *P. aeruginosa* isolates obtained from dogs with clinically diagnosed external ear canal inflammation during 2023–2024. Only one isolate per dog was included in the final dataset. The isolates were supplied by Duo-Bakt Veterinary Microbiology Laboratory. Isolation and preliminary identification were based on growth on cetrimide-containing selective medium (Biolab Zrt., Budapest, Hungary), colony morphology and pigment production. Species identification was confirmed by matrix-assisted laser desorption/ionization time-of-flight mass spectrometry (MALDI-TOF MS) before inclusion in the study. Isolates were stored at −80 °C in a Microbank system (VWR International Kft., Debrecen, Budapest) until analysis. *P. aeruginosa* ATCC 27853 was included as the quality-control/reference strain for susceptibility testing and genomic workflow control where appropriate.

### 2.2 Antimicrobial agents and MIC testing

MICs were determined by broth microdilution using cation-adjusted Mueller-Hinton broth (CAMHB; Biolab Zrt., Budapest, Hungary) and sterile 96-well polystyrene microtiter plates (VWR International Kft., Debrecen, Budapest) according to Clinical Laboratory Standards Institute (CLSI)-based dilution-testing principles. Overnight cultures were adjusted to a 0.5 McFarland standard and diluted to the working inoculum. Two-fold dilution series were prepared for ceftazidime, cefoperazone, imipenem, gentamicin, tobramycin, amikacin, polymyxin B, ciprofloxacin, enrofloxacin, marbofloxacin, norfloxacin, orbifloxacin, chlorhexidine and piperacillin-tazobactam. Plates were incubated at 37 °C for approximately 24 h. MICs were read as the lowest concentration preventing visible bacterial growth. Measurements were performed in duplicate, and discrepant results were resolved by repeat reading or repeat testing according to the laboratory protocol.

### 2.3 Breakpoints and ECOFF interpretation

MIC data were interpreted only where official or defensible interpretive criteria were available. Canine-specific breakpoints from CLSI VET01S, 7th edition (Performance Standards for Antimicrobial Disk and Dilution Susceptibility Tests for Bacteria Isolated From Animals, 2024), were used for enrofloxacin and marbofloxacin against *P. aeruginosa*. For selected antipseudomonal agents without veterinary-specific breakpoints, European Committee on Antimicrobial Susceptibility Testing (EUCAST) *Pseudomonas* spp. clinical MIC breakpoints were used for comparative interpretation. EUCAST ECOFF/tentative epidemiological cut-off value (TECOFF) values were used to classify isolates as wild-type or non-wild-type where available. For cefoperazone, norfloxacin, orbifloxacin and chlorhexidine, no formal clinical susceptibility or wild-type/non-wild-type (WT/NWT) category was assigned; these agents were summarized using MIC ranges, MIC_50_ and MIC_90_ values only. Polymyxin B was interpreted cautiously using the colistin/polymyxin framework as a comparative indicator rather than as an independently validated canine otitis breakpoint. This conservative approach was chosen to avoid inflating clinical resistance claims for agents lacking validated criteria. The complete interpretive framework applied to each tested agent is summarized in Table 1.

**Table 1:** Interpretive framework used for minimum inhibitory concentration (MIC) categorization.

Agent	Interpretive source	Threshold used	Use in analysis
Ceftazidime	EUCAST clinical breakpoint; EUCAST ECOFF	Clinical R > 8 μg/mL; NWT > 8 μg/mL	Comparative human antipseudomonal interpretation
Cefoperazone	No official validated criterion.	Not interpreted	MIC distribution only
Imipenem	EUCAST clinical breakpoint; EUCAST ECOFF	Clinical R > 4 μg/mL; NWT > 4 μg/mL	Comparative human antipseudomonal interpretation
Gentamicin	EUCAST ECOFF only	NWT > 8 μg/mL	No clinical S/I/R assigned
Tobramycin	EUCAST clinical breakpoint/ECOFF	R or NWT > 2 μg/mL	Cautious interpretation
Amikacin	EUCAST clinical breakpoint/ECOFF	R or NWT > 16 μg/mL	Cautious interpretation
Polymyxin B	Colistin/polymyxin framework	R or NWT > 4 μg/mL	Comparative proxy only
Ciprofloxacin	EUCAST clinical breakpoint/ECOFF	R or NWT > 0.5 μg/mL	Comparative human antipseudomonal interpretation
Enrofloxacin	CLSI VET canine *Pseudomonas aeruginosa*	R > 0.5 μg/mL	Veterinary clinical interpretation
Marbofloxacin	CLSI VET canine *Pseudomonas aeruginosa*	R > 0.5 μg/mL	Veterinary clinical interpretation
Norfloxacin	No official validated criterion	Not interpreted	MIC distribution only
Orbifloxacin	No official validated criterion	Not interpreted	MIC distribution only
Chlorhexidine	No antibiotic breakpoint/ECOFF	Not interpreted	Biocide MIC distribution only
Piperacillin-tazobactam	EUCAST clinical breakpoint/ECOFF	R or NWT > 16 μg/mL	Tazobactam fixed concentration assumed

R, resistant; I, intermediate; S, susceptible; NWT, non-wild-type; EUCAST, European Committee on Antimicrobial Susceptibility Testing; ECOFF, Epidemiological Cut-Off Value.

### 2.4 Whole-genome sequencing subset

**Figure 1:**
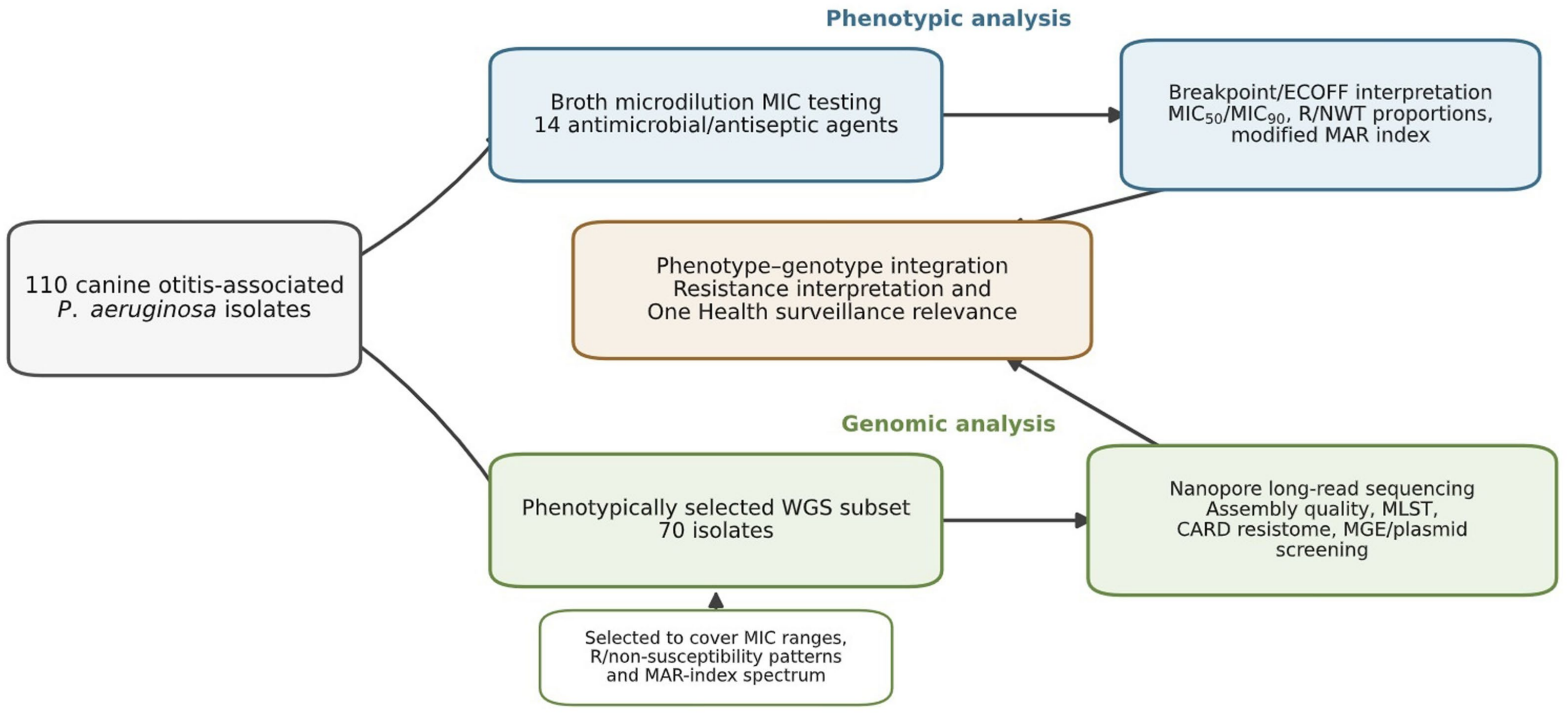
Study design and analytical workflow. The full phenotypic dataset comprised 110 canine otitis externa-associated *Pseudomonas aeruginosa* isolates subjected to broth microdilution minimum inhibitory concentration (MIC) testing against 14 antimicrobial or antiseptic agents. A phenotypically selected subset of 70 isolates, chosen to represent the major MIC ranges, resistance/non-susceptibility patterns and modified MAR-index spectrum of the full collection, underwent long-read whole-genome sequencing. Phenotypic and genomic outputs were integrated to interpret resistance patterns and their One Health surveillance relevance.

A selected subset of 70 isolates was chosen for whole-genome sequencing to capture the phenotypic diversity observed in the full 110-isolate collection. Selection was performed to include isolates with low, moderate and elevated MIC profiles across the main antimicrobial classes tested, with particular attention to fluoroquinolones, aminoglycosides, beta-lactams and polymyxin B. The subset was also checked against the full phenotypic dataset to ensure that the major MIC ranges, resistance/non-susceptibility patterns and modified MAR-index spectrum were represented. Therefore, the WGS subset was intended to provide a mechanistic genomic overview of the phenotypic diversity present in the complete collection rather than to serve as an independent random sample. An internal cross-reference table was used to link MIC isolate identifiers to the corresponding genome assembly files. The ATCC 27853 quality-control strain was excluded from the clinical WGS subset, and the downstream genomic analyses were performed on 70 clinical isolates. comprehensive antibiotic resistance database (CARD), SeqKit and CheckM records were available for all 70 selected clinical isolates, and all 70 genome assemblies were included in downstream genomic analyses. The overall study design, phenotypic dataset, WGS subset selection and analytical workflow are illustrated in Figure 1.

### 2.5 DNA extraction, library preparation and sequencing

Bacterial DNA was extracted from fresh cultures using the Quick-DNA Fungal/Bacterial Miniprep Kit (Zymo Research, Irvine, CA, USA) according to the manufacturer’s protocol. DNA concentration was measured using a Qubit 4.0 Fluorometer (Invitrogen, Life Technologies, Carlsbad, CA, USA). Libraries were prepared from 400 ng DNA using the Native Barcoding Kit 24 V14 (SQK-NBD114.24, Oxford Nanopore Technologies, Oxford, UK) and loaded on an R10.4.1 MinION flow cell. Sequencing was performed on a MinION Mk1B device for 72 h. Basecalling in super-accuracy mode and demultiplexing were performed using Dorado v7.6.8 integrated into MinKNOW v24.11.10.

### 2.6 Genome assembly and annotation

Control reads were removed using NanoLyse v1.2.1, and reads were trimmed using NanoFilt v2.8.0 by removing 50 bp from both 5-prime and 3-prime ends. Reads with mean quality scores below 8 or lengths below 500 bp were excluded. Read quality was visualized with NanoPlot v1.43.0. *De novo* assembly was performed with Flye v2.9.5-b1801. Assemblies were polished using minimap2 v2.28-r1209, racon v1.5.0 and medaka v2.0.1 with the r1041_e82_400bps_sup_v4.2.0 model. Assembly quality was assessed using SeqKit, QUAST v5.0.2, BUSCO v5.8.2 and CheckM. Sequence types were inferred using the mlst tool with the *P. aeruginosa* PubMLST seven-locus scheme. Sequence type assignment required exact allele calls for all seven housekeeping loci included in the scheme. Isolates lacking one or more complete allele calls or containing allele profiles not assignable to an existing ST in the database at the time of analysis, were reported as unassigned/incomplete allelic profiles. Antimicrobial resistance determinants were identified with ABRicate using the CARD database. Hits with at least 80% coverage and 80% nucleotide identity were retained for the primary gene-presence matrix. CARD screening was used to identify acquired and intrinsic resistance-associated determinants based on gene presence. It was not used as a comprehensive predictor of chromosomal point mutations or regulatory changes affecting quinolone resistance-determining regions, efflux regulators, *oprD*, *ampR* or other mutation-driven resistance mechanisms in *P. aeruginosa*. Antimicrobial resistance gene (ARG)-containing contigs were screened for association with mobile genetic elements and putative plasmid origin using MobileElementFinder and PlasFlow as described in the accompanying genotypic workflow.

### 2.7 Statistical analysis

MIC distributions were summarized as ranges, MIC_50_ and MIC_90_ values using the nearest-rank method. Proportions of resistant/non-susceptible and non-wild-type isolates were calculated with Wilson 95% confidence intervals. A modified multiple-antimicrobial-resistance (MAR) index was calculated for each isolate as the number of agents exceeding an official resistance/non-susceptibility threshold divided by the number of interpretable agents in the panel. This index was not calculated from all 14 tested compounds because several agents lacked valid clinical breakpoints or ECOFFs. Genome-level ARG frequencies were reported as isolate-level prevalence. Exploratory phenotype–genotype comparisons were performed descriptively; statistical testing was considered hypothesis-generating because many *P. aeruginosa* resistance determinants are core or near-core genes and because gene presence does not establish expression level or target-site mutation status.

## 3 Results

### 3.1 Phenotypic MIC distributions in the full isolate collection

**Figure 2:**
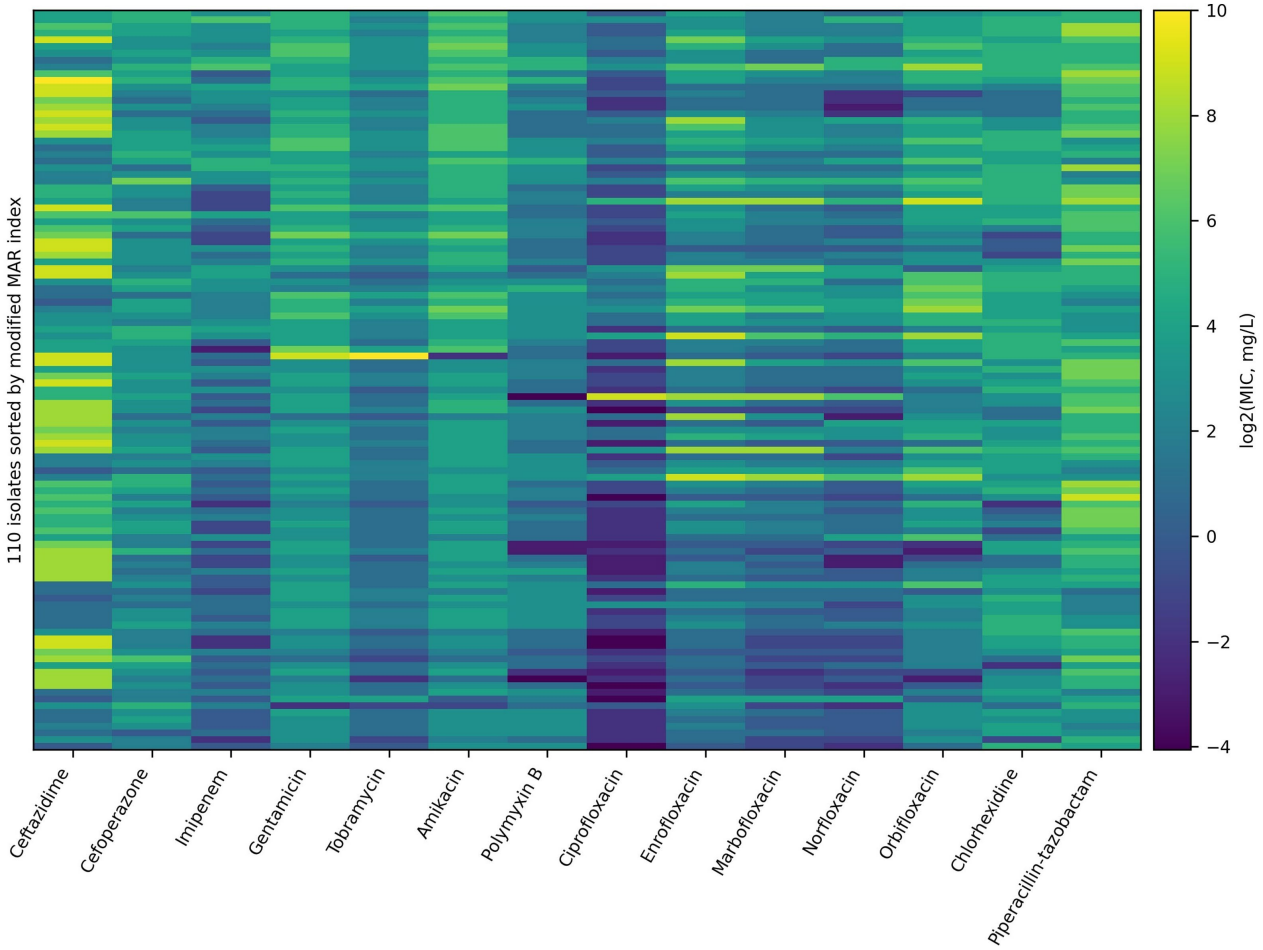
Minimum inhibitory concentration (MIC) distribution heatmap. Isolates are sorted by modified MAR index. Color intensity represents log2-transformed MIC values in μg/mL across the 14 tested agents.

All 110 isolates yielded MIC values for all 14 tested agents. The MIC distributions were broad across several antipseudomonal classes, indicating substantial phenotypic heterogeneity within the canine otitis-associated population. Ceftazidime MICs ranged from 1 to 1,024 μg/mL, with MIC_50_ and MIC_90_ values of 32 and 512 μg/mL, respectively. Piperacillin-tazobactam MICs ranged from 2 to 512 μg/mL, with MIC_50_ and MIC_90_ values of 32 and 128 μg/mL. Fluoroquinolone MICs were also widely distributed, ciprofloxacin ranged from 0.06 to 512 μg/mL, enrofloxacin from 0.5 to 512 μg/mL, marbofloxacin from 0.25 to 256 μg/mL and orbifloxacin from 0.125 to 512 μg/mL. Aminoglycoside distributions were similarly shifted, particularly for gentamicin and tobramycin. Detailed MIC ranges, MIC_50_ and MIC_90_ values, and threshold-based interpretive outcomes are shown in Table 2. The isolate-level MIC distribution across all 14 tested agents is presented as a log2-transformed heatmap in Figure 2, with isolates ordered according to the modified MAR index.

**Table 2:** Minimum inhibitory concentration (MIC) distributions and interpretive outcomes for 110 canine otitis-associated *Pseudomonas aeruginosa* isolates.

Antimicrobial	MIC range (μg/mL)	MIC_50_ (μg/mL)	MIC_90_ (μg/mL)	Clinical R/non-susceptible	EUCAST NWT
Ceftazidime	1–1,024	32	512	71/110 (64.5%; 95% CI 55.3–72.9)	71/110 (64.5%; 95% CI 55.3–72.9)
Cefoperazone	1–128	8	32	–	–
Imipenem	0.125–64	2	8	31/110 (28.2%; 95% CI 20.6–37.2)	31/110 (28.2%; 95% CI 20.6–37.2)
Gentamicin	0.25–512	16	32	–	71/110 (64.5%; 95% CI 55.3–72.9)
Tobramycin	0.25–1,024	4	8	62/110 (56.4%; 95% CI 47.0–65.3)	62/110 (56.4%; 95% CI 47.0–65.3)
Amikacin	0.25–128	16	64	41/110 (37.3%; 95% CI 28.8–46.6)	41/110 (37.3%; 95% CI 28.8–46.6)
Polymyxin B	0.06–32	4	8	43/110 (39.1%; 95% CI 30.5–48.4)	43/110 (39.1%; 95% CI 30.5–48.4)
Ciprofloxacin	0.06–512	0.5	4	45/110 (40.9%; 95% CI 32.2–50.3)	45/110 (40.9%; 95% CI 32.2–50.3)
Enrofloxacin	0.5–512	4	128	109/110 (99.1%; 95% CI 95.0–99.8)	–
Marbofloxacin	0.25–256	2	16	99/110 (90.0%; 95% CI 83.0–94.3)	–
Norfloxacin	0.125–64	2	16	–	–
Orbifloxacin	0.125–512	8	64	–	–
Chlorhexidine	0.25–32	16	32	–	–
Piperacillin-tazobactam	2–512	32	128	75/110 (68.2%; 95% CI 59.0–76.1)	75/110 (68.2%; 95% CI 59.0–76.1)

R, resistant; NWT, non-wild-type; CI, confidence interval; EUCAST, European Committee on Antimicrobial Susceptibility Testing.

**Figure 3:**
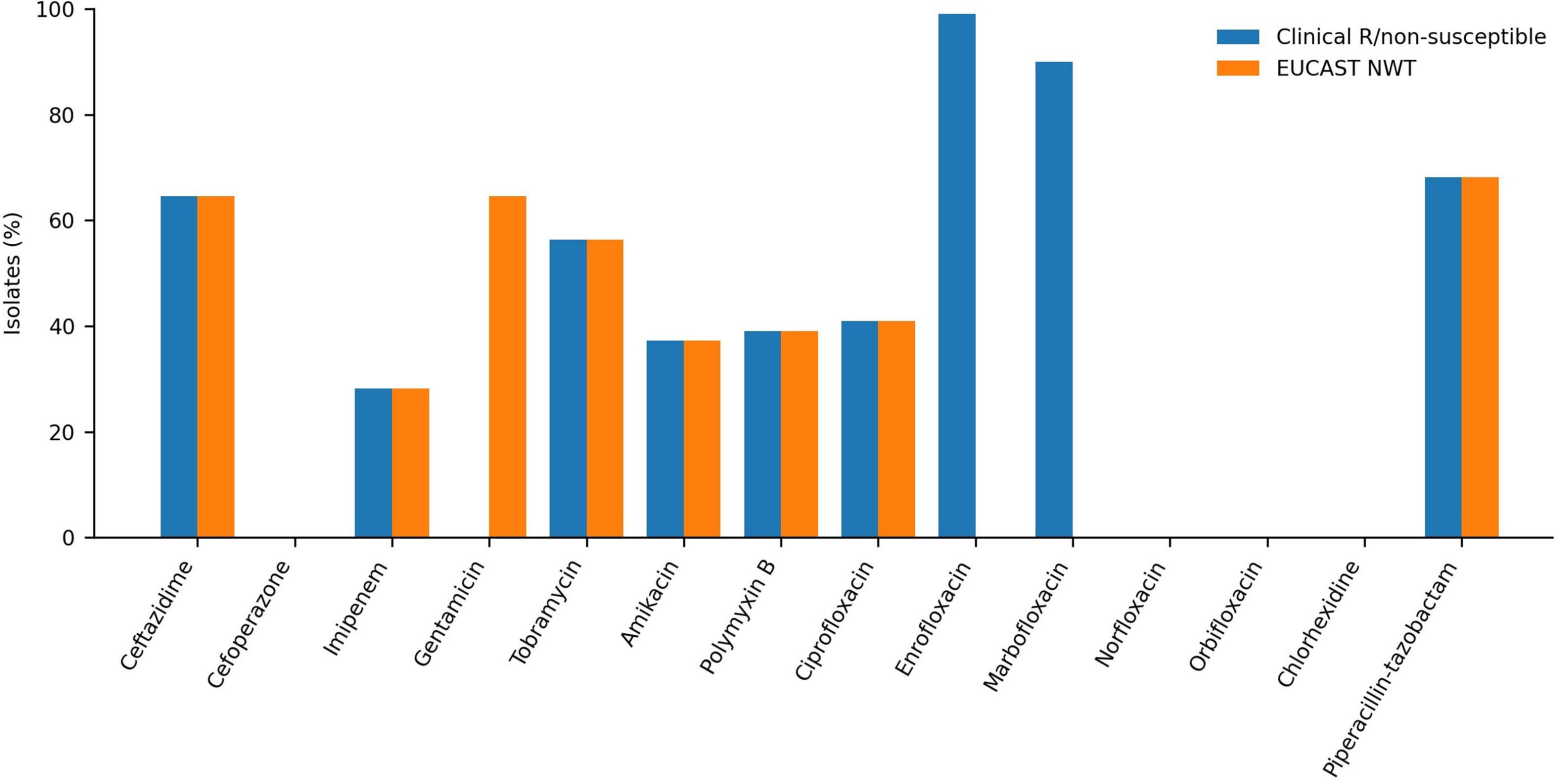
Phenotypic resistance and non-wild-type proportions. Bars show the percentage of isolates exceeding clinical resistance/non-susceptibility thresholds and European Committee on Antimicrobial Susceptibility Testing (EUCAST) epidemiological cut-off value (ECOFF)/tentative epidemiological cut-off value (TECOFF) thresholds where available. Agents lacking official interpretive criteria were not categorized.

Using the conservative interpretive framework described above, the highest clinical resistance/non-susceptibility proportions were observed for enrofloxacin (109/110; 99.1%), marbofloxacin (99/110; 90.0%), piperacillin-tazobactam (75/110; 68.2%), ceftazidime (71/110; 64.5%) and tobramycin (62/110; 56.4%). EUCAST ECOFF-based non-wild-type proportions were also high for piperacillin-tazobactam, ceftazidime, gentamicin and tobramycin. These findings indicate that a large proportion of isolates had MICs above wild-type or clinical thresholds for several systemically important antipseudomonal agents. These resistance and non-wild-type proportions are visualized in Figure 3.

### 3.2 Modified MAR index

The modified MAR index, calculated only from interpretable agents with defined resistance/non-susceptibility thresholds, ranged from 0.22 to 1.00. The mean value was 0.58, the median was 0.56 and the interquartile range was 0.44–0.67. This indicates that most isolates exceeded resistance thresholds for more than half of the interpretable antipseudomonal panel. Because intrinsic resistance and noninterpretable compounds were excluded from the denominator, this index should be interpreted as a conservative measure of acquired or clinically relevant elevated MIC burden rather than a conventional all-panel MAR score.

### 3.3 Whole-genome assembly quality and population structure

The sequence subset was designed to represent phenotypic diversity within the full MIC dataset. All 70 selected clinical genomes passed the predefined availability criteria for assembly-quality assessment, MLST assignment and CARD-based resistance determinant screening. Genome sizes were generally compatible with *P. aeruginosa*, with a mean GC content of 66.0%. Most assemblies were highly contiguous; the median number of contigs was 2.5, reflecting the long-read sequencing approach. CheckM completeness was high across the WGS subset, with a median of 99.68%. Three assemblies showed CheckM contamination values above 5%; therefore, genome-derived findings from these isolates were retained in descriptive summaries but interpreted cautiously. Summary assembly-quality metrics for the sequenced WGS subset are provided in Table 3. MLST analysis indicated a diverse population. Thirty-three isolates could not be assigned to a complete existing ST under the strict seven-locus PubMLST scheme because one or more loci lacked complete allele calls or the allelic profile did not match an existing ST at the time of analysis. Among assigned STs, ST253 was the most frequent, while ST1076, ST654, ST611, ST94 and ST200 each occurred in two isolates. The distribution of MLST calls among the 70 whole-genome-sequenced isolates is shown in Table 4.

**Table 3:** Genome assembly quality summary for the whole-genome sequencing (WGS) subset.

Metric	Value
Number of genome assemblies included in genomic analysis	70
Median contig number	2.5
Mean GC content (%)	66.00
Median CheckM completeness (%)	99.68
Median CheckM contamination (%)	0.14
Assemblies with contamination >5%	3
Selected WGS subset	70 isolates selected and included in genomic analysis

**Table 4:** Distribution of multi-locus sequence typing (MLST) calls among the 70 whole-genome-sequenced isolates.

Sequence type	Number of isolates
Unassigned/incomplete allelic profile	33
253	3
1,076	2
654	2
611	2
94	2
200	2
Other sequence types detected once	24

### 3.4 Resistance gene content in the WGS subset

CARD screening identified a broad resistance determinant background in the 70-isolate WGS subset. The most recurrent genes were dominated by intrinsic or near-core determinants, including *APH(3′)-IIb, mex*-family efflux pump components, Opr-associated outer-membrane transporters, Mux and Tri efflux-associated determinants, *arnA, basS, fosA* and *catB*-associated loci. The most frequent determinants listed in Table 5 were detected in all 70 sequenced isolates. The most frequent CARD antimicrobial resistance determinants detected in the WGS subset are summarized in Table 5. The gene spectrum is consistent with the known biology of *P. aeruginosa*, in which resistance is not primarily explained by a small number of acquired genes, but by a layered architecture of low permeability, efflux, inducible beta-lactamase activity, aminoglycoside-modifying enzymes and additional acquired or potentially mobile determinants.

**Table 5:** Most frequent comprehensive antibiotic resistance database (CARD) antimicrobial resistance determinants among the 70 whole-genome-sequenced isolates.

Gene	Isolates with gene	Prevalence (%)
*APH(3′)-IIb*	70	100.0
*armR*	70	100.0
*mexA*	70	100.0
*mexB*	70	100.0
*mexF*	70	100.0
*mexE*	70	100.0
*mexD*	70	100.0
*mexC*	70	100.0
*muxA*	70	100.0
*muxC*	70	100.0
*oprJ*	70	100.0
*oprN*	70	100.0
*opmB*	70	100.0
*opmH*	70	100.0
*oprM*	70	100.0
*opmD*	70	100.0
*mexI*	70	100.0
*mexJ*	70	100.0
*mexK*	70	100.0
*mexH*	70	100.0

**Figure 4:**
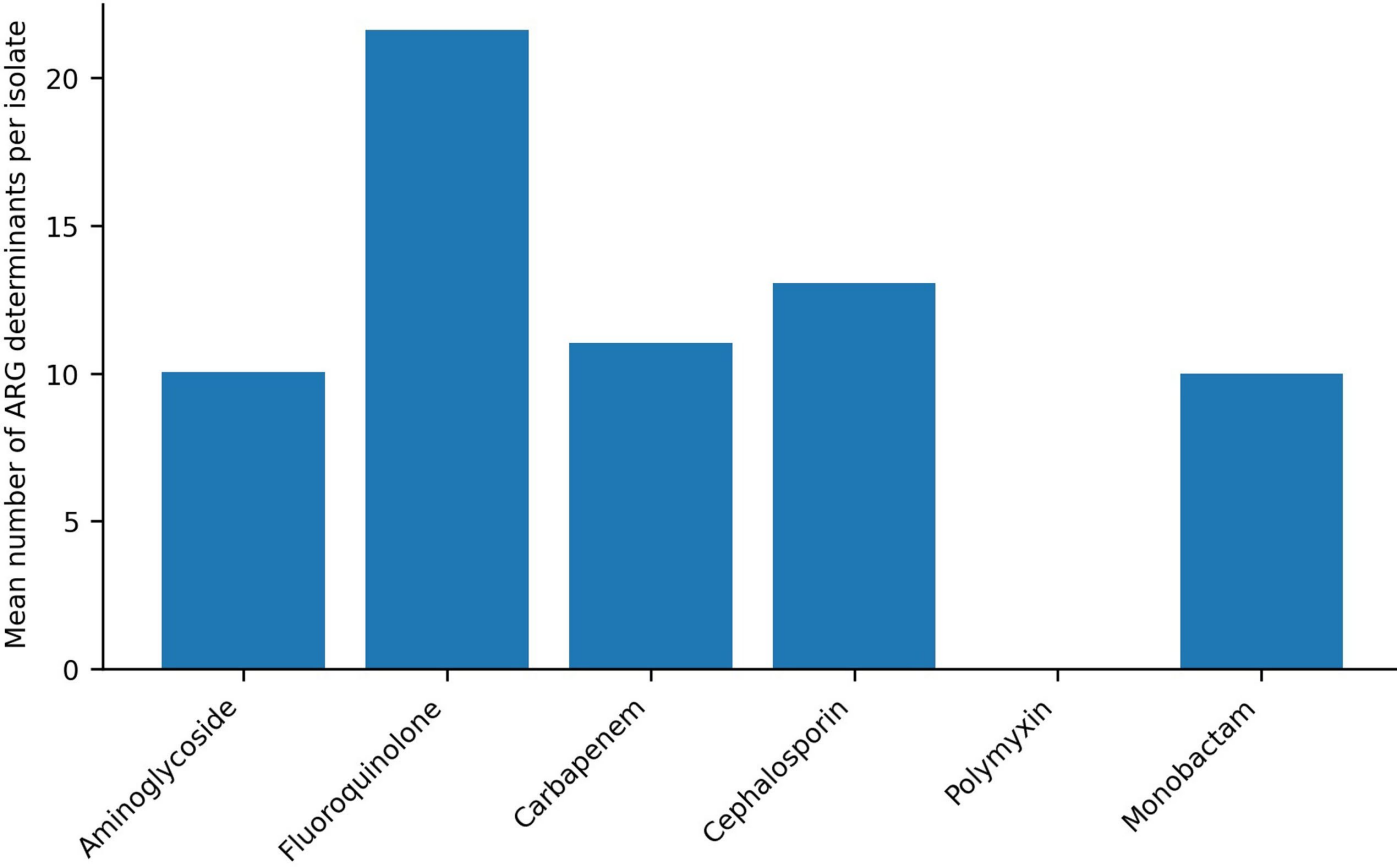
Class-level burden of predicted resistance determinants. Mean numbers of comprehensive antibiotic resistance database (CARD) determinants per isolate are shown by antimicrobial class. This descriptive measure reflects genomic resistance architecture rather than direct clinical resistance prediction.

Beta-lactam-associated determinants included PDC and OXA variants, while fluoroquinolone-associated determinants included efflux systems and *crpP* in a subset of isolates. The presence of *crpP* is particularly relevant because it may contribute to reduced fluoroquinolone activity and, when plasmid-associated, has higher horizontal transfer significance. Aminoglycoside-associated genes were dominated by *APH(3′)-IIb* and related modifying-enzyme or efflux components. However, the high prevalence of core resistance-associated genes means that MIC elevation cannot be inferred from gene presence alone. The high fluoroquinolone MICs observed in the phenotypic dataset are compatible with the possible involvement of quinolone resistance-determining region mutations in *gyrA*, *gyrB*, *parC* and *parE*, in addition to efflux-associated mechanisms. However, targeted mutation calling was not included in the present analysis; therefore, the study does not assign fluoroquinolone resistance to specific QRDR mutations. This limitation prevents deterministic phenotype–genotype prediction and supports interpreting the WGS results as a resistance-associated genomic background rather than as a complete mechanistic explanation. Similarly, elevated ceftazidime, piperacillin-tazobactam and imipenem MICs may reflect a combination of chromosomal beta-lactamase background, regulatory effects, efflux activity and porin-related mechanisms, including possible *oprD*-associated changes. Because targeted *oprD*, *ampC*/*ampR* and beta-lactamase regulatory mutation analysis was not performed, beta-lactam and carbapenem MICs were not attributed to individual chromosomal mutations. The class-level burden of predicted resistance determinants is shown descriptively in Figure 4.

### 3.5 Phenotype–genotype integration

The integrated dataset supports a model in which elevated MICs in canine otitis-associated *P. aeruginosa* arise from the cumulative effect of intrinsic resistance architecture, possible acquired determinants and likely target/regulatory mutations. The high MICs to fluoroquinolones, aminoglycosides and beta-lactams were directionally consistent with the frequent detection of efflux-associated determinants, aminoglycoside-modifying enzymes and beta-lactamase genes. Nevertheless, formal phenotype–genotype prediction was limited by the high background prevalence of many CARD hits and by the lack of expression and mutation-level data in the analysis. The most defensible interpretation is therefore mechanistic rather than deterministic: the WGS data indicate a resistance-associated genomic background, whereas MIC testing remains essential for clinical decision-making.

## 4 Discussion

This study provides an integrated phenotypic and genomic assessment of canine otitis externa-associated *P. aeruginosa*, combining MIC data from 110 clinical isolates with WGS-based resistance determinant screening in a phenotypically selected 70-isolate subset. The central finding is that the collection showed markedly elevated MICs across several antipseudomonal classes, most prominently fluoroquinolones, selected aminoglycosides, ceftazidime, piperacillin-tazobactam and imipenem. This pattern places the present Hungarian collection at the more resistant end of the spectrum reported for canine otitis-associated *P. aeruginosa*, although direct numerical comparison must be made cautiously because published studies differ in geography, sampling period, disease severity, prior antimicrobial exposure, antimicrobial panels, test methodology and interpretive criteria (1, 16, 17, 24–26). Similar challenges in comparing antimicrobial susceptibility profiles across host species and study designs have been noted in recent veterinary susceptibility studies from Hungary (27).

The most prominent phenotypic finding was the high frequency of resistance/non-susceptibility to the veterinary fluoroquinolones. In our dataset, 109/110 isolates (99.1%) were categorized as resistant or non-susceptible to enrofloxacin and 99/110 (90.0%) to marbofloxacin using the selected interpretive framework. These proportions are substantially higher than those reported in several previous canine otitis studies. In the Croatian study by Mekic et al., *P. aeruginosa* isolates from canine otitis showed lower resistance levels to the antipseudomonal agents tested, including fluoroquinolones (16). McKay et al. tested 100 *P. aeruginosa* isolates from canine otitis and concluded that marbofloxacin had better *in vitro* activity than enrofloxacin or orbifloxacin (28). A more recent Midwestern United States study reported lower susceptibility to fluoroquinolones than to aminoglycosides, with susceptibility of 67% to marbofloxacin and only 32% to enrofloxacin among 216 *P. aeruginosa* otitis isolates (29). Likewise, Secker et al. reported enrofloxacin resistance in 25.4% of 252 European canine otitis isolates, which is clinically relevant but still far below the level observed in the present collection (24). This discrepancy may reflect enrichment of the present collection for difficult-to-treat or recurrent cases, differences in local antimicrobial exposure, or methodological differences in isolate selection and interpretive criteria.

The ciprofloxacin results further support the interpretation that fluoroquinolone resistance is a major feature of this collection. We observed ciprofloxacin resistance/non-wild-type status in 45/110 isolates (40.9%). This is lower than the enrofloxacin and marbofloxacin rates but still clinically important, especially because ciprofloxacin is widely used in human medicine and is commonly used as a comparator for antipseudomonal fluoroquinolone activity. The lower ciprofloxacin resistance rate relative to enrofloxacin and marbofloxacin is consistent with earlier observations that individual fluoroquinolones may differ in their *in vitro* activity against canine *P. aeruginosa* isolates (28, 30). Mechanistically, fluoroquinolone resistance in *P. aeruginosa* is rarely explained by a single determinant; it typically reflects QRDR mutations in *gyrA*, *gyrB*, *parC* and *parE*, efflux pump activity, and sometimes plasmid-associated or chromosomal modifiers such as *crpP*. Tejedor et al. showed that canine otitis-derived fluoroquinolone-resistant *P. aeruginosa* commonly harbored *gyrA* mutations and efflux overexpression (31), while Arais et al. and Park et al. further documented the relevance of *gyrA/gyrB* or *gyrA/parC* mutations in companion-animal *P. aeruginosa* (32, 33). Therefore, the CARD-based detection of efflux-associated determinants and *crpP* in the present WGS subset should be interpreted as part of a broader resistance architecture rather than as a complete explanation of the observed MICs.

Aminoglycoside findings were also less favorable than those reported in several external datasets. In our full MIC collection, 71/110 isolates (64.5%) were non-wild-type for gentamicin, 62/110 (56.4%) for tobramycin and 41/110 (37.3%) for amikacin. By comparison, the Japanese study by Elfadadny et al. reported resistance rates of only 10.3% to gentamicin and 10.3% to tobramycin among 29 canine ear canal isolates, and considered tobramycin and gentamicin among the agents retaining useful *in vitro* activity (17). Secker et al. found tobramycin to be the most active antimicrobial in their European canine otitis collection, with 99.6% susceptibility (24). KuKanich et al. similarly reported higher susceptibility to aminoglycosides, with 82% susceptibility to gentamicin and 81% to amikacin in canine otitis isolates (29). Earlier Hungarian work by Jerzsele and Pasztine-Gere examined 68 canine otitis-associated *P. aeruginosa* isolates and reported broad MIC ranges for both marbofloxacin and gentamicin but also demonstrated that the marbofloxacin-gentamicin combination reduced MIC values and produced partial synergistic/additive effects in many isolates (34). Against this background, the aminoglycoside MIC profile observed here is concerning and strengthens the argument that topical or systemic therapy should not be selected empirically in chronic *Pseudomonas* otitis.

The elevated ceftazidime, imipenem and piperacillin-tazobactam MICs require a different clinical interpretation from the veterinary fluoroquinolone and aminoglycoside results. These agents are not routine first-line options for canine otitis externa, and carbapenems in particular should be regarded as critically important human medicines rather than ordinary veterinary therapeutic tools. Nevertheless, their inclusion is epidemiologically informative. In this collection, 71/110 isolates (64.5%) exceeded the ceftazidime threshold, 75/110 (68.2%) exceeded the piperacillin-tazobactam threshold and 31/110 (28.2%) exceeded the imipenem threshold. These rates are much higher than the Japanese study, where only 3.4% of isolates were resistant to ceftazidime, the isolates were susceptible to piperacillin-tazobactam, and imipenem/meropenem resistance was 3.4% (17). Secker et al. reported imipenem resistance in 8.3% and meropenem resistance in 0.79% of European canine otitis isolates (24). Published reports of VIM-2-producing *P. aeruginosa* from dogs with pyoderma and otitis in Korea demonstrate that carbapenemase-producing veterinary isolates can occur, but they remain exceptional events rather than the usual explanation for carbapenem non-susceptibility (35). Consequently, the imipenem signal in the present material should be discussed primarily as a One Health warning and as a marker of possible porin/efflux/beta-lactamase-mediated reduced susceptibility, not as a justification for carbapenem use in companion animals.

The polymyxin B results should also be interpreted with restraint. In the absence of a direct, canine otitis-specific clinical breakpoint for polymyxin B, the present analysis used a colistin/polymyxin interpretive proxy and treated the result primarily as an epidemiological signal. The finding that 43/110 isolates (39.1%) exceeded the selected threshold suggests that reduced susceptibility to membrane-active last-resort or topical agents may be present in part of the collection. However, polymyxin activity in otitis externa is influenced by topical formulation, local concentration, organic load, pH, biofilm state and ear cleaning, none of which is captured by planktonic MIC testing alone. This is particularly important because *Pseudomonas* otitis is frequently biofilm-associated, and biofilm can markedly alter antimicrobial tolerance even without stable inherited resistance (1, 11, 24, 36).

The modified MAR index complements the single-agent analysis by summarizing the cumulative resistance burden. Because we calculated this index only from agents with defensible interpretive thresholds, it is deliberately conservative and does not artificially inflate resistance by including cefoperazone, norfloxacin, orbifloxacin or chlorhexidine where no official clinical breakpoint or ECOFF was available. The high mean MAR index observed in the dataset is therefore not a statistical artifact of over-interpreting the entire MIC panel but reflects repeated threshold exceedance across multiple clinically or epidemiologically relevant antipseudomonal classes. This is consistent with the principle that MDR categorization in veterinary pathogens should be based on acquired non-susceptibility across antimicrobial classes rather than on intrinsic resistance or uninterpretable compounds (13, 15).

The genomic results help explain why phenotype–genotype correspondence in *P. aeruginosa* is inherently complex. CARD screening identified a broad background of conserved intrinsic resistance determinants, including RND efflux systems, outer-membrane components, chromosomal beta-lactamase-related determinants and aminoglycoside-associated determinants. Similar observations were reported by Secker et al., who found that canine otitis isolates had relatively consistent AMR gene complements despite variation in phenotypic susceptibility (24). This is a key point for the Discussion: the presence of Mex, Opr, PDC, OXA or APH-family determinants in *P. aeruginosa* does not automatically predict a resistant MIC. Expression level, regulatory mutations, target-site mutations, porin loss, biofilm state, local environment and antimicrobial exposure history are all decisive (10, 12, 18, 21). Therefore, the present dataset should not be presented as a simple gene-presence/resistance model. Its strength is instead the parallel documentation of high MIC burden and a genomic background compatible with multiple overlapping resistance mechanisms.

The detection of mobilization-associated signals, including plasmid-predicted or mobile-element-proximal resistance determinants in part of the WGS subset, is important but should not be overinterpreted. In *P. aeruginosa*, much clinically important resistance is chromosomal and mutation-driven, yet mobile genetic elements can contribute to clinically relevant resistance and facilitate One Health dissemination. The Japanese canine otitis study emphasized the potential relevance of dog-associated *P. aeruginosa* as a reservoir of resistant lineages and identified sequence types related to human clinical isolates (17). Secker et al. similarly identified ST111 and ST244 among canine otitis isolates, both recognized as high-risk sequence types in human nosocomial infection contexts (24). In addition, two isolates in the present WGS subset were assigned to ST654. This is noteworthy because ST654 has been described among international high-risk *P. aeruginosa* clones associated with multidrug resistance and healthcare-associated infections, including reports of extensively drug-resistant ST654 carrying carbapenemase genes. Although the detection of ST654 in only two canine otitis isolates does not by itself indicate transmission between human and veterinary settings, it supports the value of comparing companion-animal clinical isolates with human and environmental genomic surveillance datasets (37, 38). The present dataset also contains several typed genomes and many untyped or incomplete MLST calls, which means that the population structure should be treated as preliminary until the assembly set is fully curated. Nonetheless, the combined phenotypic and genomic findings support continued surveillance of canine otitis-associated *P. aeruginosa* at the companion animal-human-environment interface. This One Health interpretation is also supported by recent Hungarian work highlighting wildlife and environmental interfaces as relevant reservoirs and dissemination routes for antimicrobial resistance (39). The relevance of jointly evaluating phenotypic resistance, resistance genes and mobile genetic elements has also been demonstrated in recent farm-animal *E. coli* studies (40).

From a One Health surveillance perspective, the present findings should be interpreted as a national companion-animal clinical dataset rather than as evidence of broad epidemiological dissemination. Nevertheless, canine otitis-associated *P. aeruginosa* isolates are relevant to integrated AMR surveillance because they originate from a setting characterized by close human–animal contact, repeated antimicrobial exposure and frequent outpatient antimicrobial use. The detection of high MIC burdens to fluoroquinolones, aminoglycosides and antipseudomonal beta-lactams, together with resistance-associated genomic determinants and sequence types of potential epidemiological interest, supports the value of including companion-animal clinical isolates in national and regional AMR monitoring frameworks. Such datasets can complement human hospital, livestock and environmental surveillance by enabling cross-sectoral comparison of resistance phenotypes, sequence types, resistance determinants and mobile-element signals.

There are several limitations that should be explicitly acknowledged. First, the 70-genome subset was selected to represent phenotypic diversity and is not a random subset of the 110 MIC-tested isolates; therefore, gene prevalence in the WGS subset should not be generalized directly to all canine otitis isolates. However, because the subset was selected to cover the main MIC ranges, resistance/non-susceptibility profiles and modified MAR-index spectrum observed in the full collection, it provides a suitable genomic framework for interpreting the phenotypic diversity detected in this study. Second, some comparisons with the literature are constrained by differences between MIC and disk diffusion testing, veterinary versus human breakpoints, CLSI versus EUCAST interpretive rules, and the changing definitions of susceptible, intermediate, susceptible-dose dependent and non-wild-type categories. Third, phenotypic MIC testing was performed under planktonic *in vitro* conditions and does not reproduce the inflamed, exudative, biofilm-rich and formulation-dependent environment of the canine ear canal. Fourth, the genomic analysis was based primarily on resistance determinant screening and did not include targeted mutation calling in QRDR loci, efflux regulatory genes, *oprD*, *ampC*/*ampR* or other chromosomal loci known to influence *P. aeruginosa* resistance. Consequently, phenotype–genotype associations were interpreted mechanistically and descriptively rather than as deterministic predictions. These limitations do not weaken the main conclusion, but they define its proper scope: this is a laboratory-based susceptibility and genomic resistance study, not a direct clinical outcome trial.

Clinically, the findings reinforce the need for microbiology-guided management of canine *Pseudomonas* otitis. The high fluoroquinolone and aminoglycoside MIC burden argues against repeated empirical recycling of the same topical or systemic antimicrobials without culture and susceptibility testing. At the same time, the data should not be interpreted as supporting escalation to critically important antimicrobials primarily reserved for human medicine. Instead, the practical implications are improved sampling, ear cleaning, evaluation of otitis media and tympanic integrity, appropriate topical formulation, avoidance of unnecessary systemic treatment, and rational use of combination or adjuvant strategies where supported by evidence. The earlier Hungarian marbofloxacin-gentamicin synergy work and the broader literature on Tris-EDTA and biofilm-disrupting approaches suggest that future studies should evaluate whether local combination strategies can overcome some of the high planktonic MICs observed here without increasing selection pressure for systemically important agents (1, 34, 41, 42). The importance of antimicrobial-use monitoring and increased reliance on bacteriological and susceptibility testing has also been emphasized in recent Hungarian veterinary stewardship studies (43).

Overall, the present study extends previous canine *P. aeruginosa* otitis work by combining a relatively large MIC-tested clinical collection with WGS-based resistance determinant screening in a selected subset. Compared with recent Japanese, European, Croatian, Brazilian and North American datasets, the Hungarian isolates show an unusually high resistance burden for several clinically relevant or epidemiologically important antipseudomonal agents. The most defensible interpretation is not that a single resistance mechanism dominates, but that chronic canine otitis-associated *P. aeruginosa* in this collection carries a multifactorial resistance phenotype shaped by intrinsic resistance, efflux biology, beta-lactamase background, target modification, possible mobile elements and local antimicrobial selection pressure. This makes the collection clinically relevant for veterinary dermatology and epidemiologically relevant for One Health AMR surveillance.

## 5 Conclusion

Canine otitis-associated *P. aeruginosa* isolates in this collection showed broad MIC distributions and frequent elevated MICs to several clinically relevant antipseudomonal agents. The WGS subset revealed a resistance-associated genomic background dominated by efflux, beta-lactamase, aminoglycoside-modifying and membrane-modification determinants. MIC testing remains indispensable for therapeutic decision-making, while WGS provides mechanistic and epidemiological context. The findings support routine culture- and susceptibility-guided management of *Pseudomonas* otitis and reinforce the need for companion-animal AMR surveillance within a One Health framework.

## Data Availability

The genome sequence data generated in this study have been deposited in the National Center for Biotechnology Information (NCBI) database under BioProject accession number PRJNA1475700 and BioSample accession numbers SAMN57946491–SAMN57946560. The phenotypic MIC dataset, isolate-level metadata and additional analysis outputs are provided as supplementary material.
